# Tuberculosis of Gall Bladder Presenting as Empyema

**DOI:** 10.4103/1319-3767.39630

**Published:** 2008-04

**Authors:** Leo F. Tauro, John JS Martis, Hejmadi D. Shenoy

**Affiliations:** Department of General Surgery, Fr. Muller Medical College Hospital, Kankanady, Mangalore - 2, Karnataka, India. E-mail: drlftauro@rediffmail.com

Sir,

Tuberculosis of the gall bladder is extremely rare, and hence, not adequately documented.[1] Cholelithiasis is a common association of both tuberculosis and malignancy of the gall bladder. Their clinical presentations also mimic each other.[2]

A 58-year-old male patient presented with a year-long complaint of chronic upper abdominal pain along with fever since the last 5 days. There was no prior history of tuberculosis or of contact with such patients. Clinically, he was febrile and the abdomen showed epigastric and right hypochondriac tenderness. Murphy's sign was positive. Hematological examination revealed leucocytosis and neutrophilia. ESR was 67 mm in the first hour. Liver enzymes and chest X-ray were normal. Abdominal ultrasound revealed thickened wall with distended gall bladder and a calculus in the neck of the gall bladder suggestive of chronic calculous cholecystitis.

After 3 days of failed conservative treatment, laparotomy through Kocher's incision revealed thickened, distended, and densely adherent gall bladder with omental adhesions. Pus was aspirated from the tense gall bladder. Following adhesiolysis, cholecystectomy was performed by the fundus-first method. Histopathological examination of the specimen revealed features suggestive of acute on chronic cholecystitis with chronic granulomatous reaction and foreign body giant cells. Serum IgG for ***Mycobacterium tuberculosis*** was highly positive (H: 1359); whereas, IgM was negative. Sputum was negative for acid-fast bacilli. In view of the positive serological test for tuberculosis, a complete course of antitubercular treatment was administered for 7 months (four drugs—INH, rifampicin, ethambutol, and pyrazinamide—for 2 months and two drugs—INH, rifampicin—for the next 5 months). He remained asymptomatic after 1 year of follow-up.

In English literature, >50 cases of tuberculosis of the gall bladder have been reported.[1] Rarity of tubercular involvement of the gall bladder has been attributed to high alkalinity of bile and bile acids inhibiting the growth of tubercle bacillus. It has been suggested that cystic duct obstruction leads to disappearance of bile acids from the gall bladder, which lowers the resistance to this infection. Previous damage to the gall bladder due to gallstones seems to be a prerequisite for the development of tuberculous cholecystitis as almost all reported cases have co-existent gallstones. Literature review revealed only three case reports of tuberculous cholecystitis without associated gall stones or cystic/common bile duct obstruction.[2]

Schistosomal cholecystitis is a rare variety of granulomatous infection and must be distinguished from the tuberculous form. In the medical literature, >10 cases of schistosomal cholecystitis are described.[3] Sharara ***et al.***[3] described a case of acute granulomatous schistosomal acalculous cholecystitis.

As tuberculous cholecystitis is difficult to diagnose preoperatively, all resected cholecystectomy specimens should be sent for confirmatory histopathological examination, especially in endemic areas where the diagnosis is suspected. In our case, there were dense adhesions around the gall bladder with signs of acute inflammation suggestive of acute on chronic cholecystitis. Moreover, intraoperatively, the gall bladder was found to contain pus and stones at the neck. Surgery was justified in our case since there was co-existent empyema of the gall bladder. A study by Raja ***et al.***[4] showed that the sensitivities of IgG, IgA, and IgM antibodies for tuberculosis are 62, 52, and 11%, respectively, while the specificities are is 100, 97, and 95%, respectively. Kaustova ***et al.***[5] reported that the remarkable sensitivity of the serological tests applied to culture-negative pulmonary and nonpulmonary tuberculosis makes the test a good adjuvant in cases of suspicion of tuberculosis.

Although tuberculosis of the gall bladder is a rare entity, we recommend subjecting all resected gall bladder specimens for histopathological examination in endemic areas to rule out tuberculosis.
